# CHD3 Proteins and Polycomb Group Proteins Antagonistically Determine Cell Identity in Arabidopsis

**DOI:** 10.1371/journal.pgen.1000605

**Published:** 2009-08-14

**Authors:** Ernst Aichinger, Corina B. R. Villar, Sara Farrona, José C. Reyes, Lars Hennig, Claudia Köhler

**Affiliations:** 1 Department of Biology and Zurich-Basel Plant Science Center, Swiss Federal Institute of Technology, ETH Centre, Zurich, Switzerland; 2 Max Planck Institute for Plant Breeding Research, Cologne, Germany; 3 Centro Andaluz de Biología Molecular y Medicina Regenerativa, Consejo Superior de Investigaciones Científicas, Sevilla, Spain; The University of North Carolina at Chapel Hill, United States of America

## Abstract

Dynamic regulation of chromatin structure is of fundamental importance for modulating genomic activities in higher eukaryotes. The opposing activities of Polycomb group (PcG) and trithorax group (trxG) proteins are part of a chromatin-based cellular memory system ensuring the correct expression of specific transcriptional programs at defined developmental stages. The default silencing activity of PcG proteins is counteracted by trxG proteins that activate PcG target genes and prevent PcG mediated silencing activities. Therefore, the timely expression and regulation of PcG proteins and counteracting trxG proteins is likely to be of fundamental importance for establishing cell identity. Here, we report that the chromodomain/helicase/DNA–binding domain CHD3 proteins PICKLE (PKL) and PICKLE RELATED2 (PKR2) have trxG-like functions in plants and are required for the expression of many genes that are repressed by PcG proteins. The *pkl* mutant could partly suppress the leaf and flower phenotype of the PcG mutant *curly leaf*, supporting the idea that CHD3 proteins and PcG proteins antagonistically determine cell identity in plants. The direct targets of PKL in roots include the PcG genes *SWINGER* and *EMBRYONIC FLOWER2* that encode subunits of Polycomb repressive complexes responsible for trimethylating histone H3 at lysine 27 (H3K27me3). Similar to mutants lacking PcG proteins, lack of PKL and PKR2 caused reduced H3K27me3 levels and, therefore, increased expression of a set of PcG protein target genes in roots. Thus, PKL and PKR2 are directly required for activation of PcG protein target genes and in roots are also indirectly required for repression of PcG protein target genes. Reduced PcG protein activity can lead to cell de-differentiation and callus-like tissue formation in *pkl pkr2* mutants. Thus, in contrast to mammals, where PcG proteins are required to maintain pluripotency and to prevent cell differentiation, in plants PcG proteins are required to promote cell differentiation by suppressing embryonic development.

## Author Summary

In higher eukaryotes only a small proportion of genomic information is required in any specific cell type at a given developmental stage. The intricate decision whether a gene should be active or repressed is made by the counteractive activities of trithorax group (trxG) and Polycomb group (PcG) proteins that form part of a chromatin-based cellular memory system. Here we show that the CHD3 proteins PICKLE and PICKLE RELATED2 (PKR2) have trxG-like functions in plants and activate PcG protein target genes. Lack of PKL function can partially suppress PcG mutant leaf and flower phenotypes, supporting the idea that CHD3 proteins and PcG proteins act antagonistically during plant development. We identified PcG genes among the direct PKL/PKR2 targets in roots and demonstrated that lack of *pkl pkr2* results in reduced PcG protein activities, leading to similar root phenotypes in *pkl pkr2* and PcG protein mutants. Previous studies have implicated PKL as a transcriptional repressor, but we provide evidence that CHD3 proteins such as PKL and PKR2 act as transcriptional activators in plants and assume trxG-like function to counteract PcG protein–mediated gene repression.

## Introduction

Dynamic regulation of chromatin structure is the underlying scheme for modulating genome activities in higher eukaryotes. There are two major classes of proteins with enzymatic activities directed at chromatin - histone modifying enzymes and ATP dependent chromatin remodelers. Histone modifying enzymes add or remove posttranslational modifications such as acetylation, methylation, phosphorylation and ubiquitinylation on histones. These modifications are recognized and bound by factors that cause changes in chromatin structure by not well understood mechanisms [1]. ATP dependent chromatin remodelers modify chromatin structure by altering interactions between DNA and histone octamers, resulting in changes of nucleosome position or composition associated with changes in nucleosomal DNA accessibility [2].

CURLY LEAF (CLF) and PICKLE (PKL) are examples of these two enzyme classes in plants. CLF is a Polycomb group (PcG) protein with histone methyltransferase activity [3],[4], and PKL is a predicted ATP-dependent chromatin remodeling factor of the chromodomain/helicase/DNA-binding domain (CHD3) subfamily [5],[6].

Members of the CHD3 subfamily are characterized by the presence of two tandemly arranged chromodomains as well as the presence of one or two PhD (plant-homeo-domain) zinc fingers preceding the chromodomains [7]. CHD3 family members of flies and mammals are part of the NuRD (nucleosome remodeling and deacetylase) multiprotein complex that is implicated to couple ATP-dependent chromatin remodeling and deacetylation resulting in transcriptional repression [7]. However, several studies also implicate a function of CHD3 family members in transcriptional activation [8]–[10].

CLF is a homolog of the metazoan SET domain protein Enhancer of zeste, and similar to animal PcG proteins CLF is part of a multiprotein Polycomb repressive complex 2 (PRC2)-like complex that trimethylates histone H3 on lysine 27 (H3K27me3) [4],[11],[12]. This modification is recognized by the chromodomain containing protein LIKE HETEROCHROMATIN PROTEIN 1 (LHP1) that together with the RING finger domain proteins AtRING1a and AtRING1b causes gene repression by not yet understood mechanisms [13]–[15]. Lack of CLF function causes reduced H3K27me3 levels associated with pleiotropic developmental aberrations like formation of curled leaves, homeotic transformations of flowers and early flowering [4],[16],[17]. CLF acts partially redundant with its homolog SWINGER (SWN), and lack of both proteins causes cells to de-differentiate and to form callus-like tissues that give rise to somatic embryos [18]. Similarly, lack of PKL function causes derepression of embryogenic traits in seedling roots, accumulation of seed storage reserves and formation of somatic embryos; albeit this pickle root phenotype only occurs with very low penetrance [19]. The embryonic master regulator gene *LEAFY COTYLEDON1* (*LEC1*) is activated in both, *pkl* and *clf swn* double mutants [6],[20]. Overexpression of *LEC1* causes somatic embryogenesis [21],[22], suggesting that *LEC1* is critically responsible for somatic embryogenesis in *pkl* and *clf swn* mutants. Thus, lack of PcG proteins CLF and SWN as well as lack of PKL causes cell dedifferentiation and somatic embryogenesis, however, the underlying molecular mechanisms for this common phenotype remain unclear. Recent studies observed an overlap of genes being up-regulated in *pkl* mutants and genes enriched for H3K27me3, suggesting a functional connection of PKL and PcG pathways [23]. This idea was supported by the finding that lack of PKL caused reduced H3K27me3 levels at selected loci, whereas histone acetylation levels remained largely unaffected in *pkl* mutants [23]. Thus it seemed unlikely that PKL is part of a NuRD-like complex in plants but rather assumes an as yet unidentified role in gene regulation.

We set out to identify the functional connection of PKL and PcG proteins and found that in contrast to its anticipated role as a repressor, PKL has trithorax group (trxG)-like functions and is required for the activation of PcG target genes. Among its direct targets we identified the genes for the PcG proteins SWN and EMF2. Lack of PKL as well as its close homolog PKR2 caused reduced expression of genes for PcG proteins in primary roots, concomitantly with reduced H3K27me3 levels. This in turn is likely responsible for increased *LEC1* expression and derepression of embryonic traits in *pkl pkr2* primary roots. Expression of PcG genes is independent of PKL in aerial plant tissues and *pkl* can partly suppress the *clf* leaf and flower phenotype, supporting the idea that PKL and PcG proteins antagonistically determine cell identity in plants.

## Results

### PICKLE and PICKLE RELATED2 Act Redundantly in Suppressing Embryonic Identity

**Figure 1:**
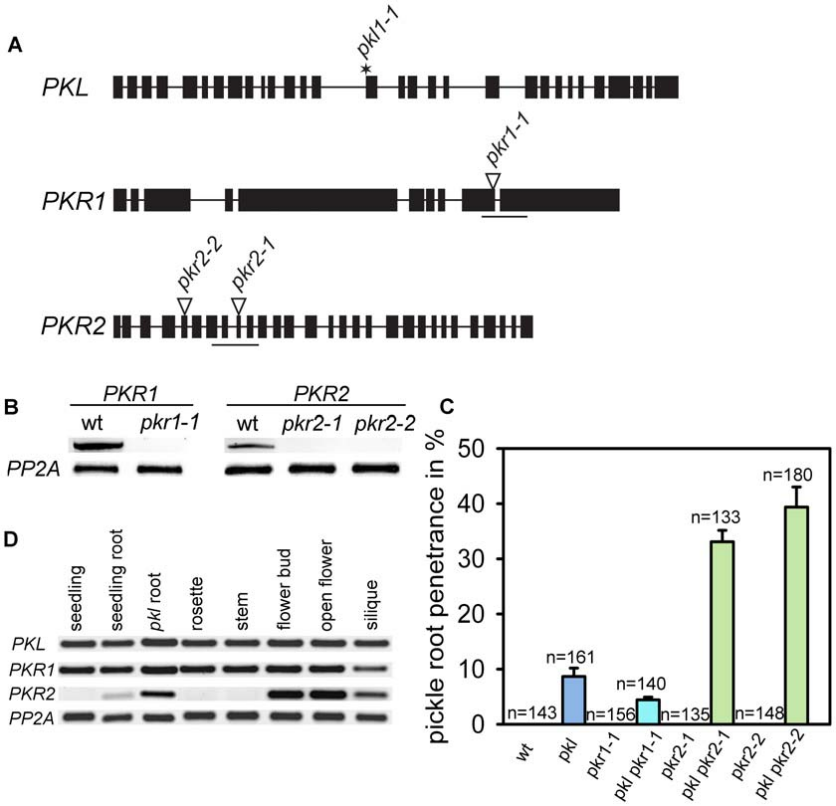
PKL and PKR2 act redundantly to maintain root cell identity. (A) Genomic organization of the *PKL*, *PKR1* and *PKR2* loci. The *pkl-1* mutation (asterisk) is a described EMS allele cit_bf [32]cit_af ref_bf(Li, 2005 ref_num5603)ref_af. T-DNAs (black triangles) are inserted in the 9th exon in the *pkr1-1* and in the 9^th^ and 5^th^ exons in *pkr2-1* and *pkr2-2* mutants, respectively. Black boxes represent exons, connecting lines introns. Analyzed regions tested by RT-PCR are marked by a line below the genomic loci. (B) Transcript accumulation in wild-type *pkr1-1*, *pkr2-1*, and *pkr2-2* flower buds was tested by RT-PCR. (C) pickle root formation was assayed in five-day-old wild-type, *pkl*, *pkr1-1*, *pkl pkr1-1*, *pkr2-1*, *pkl pkr2-1*, *pkr2-2*, and *pkl pkr2-2* seedling roots. Numbers on top of bars represent total number of scored seedlings. Experiments were performed in triplicates. Error bars, SEM. (D) RT–PCR analysis of *PKL*, *PKR1* and *PKR2* in different plant tissues. wt, wild-type.

Investigations of the underlying molecular mechanism of the pickle root phenotype have been hampered by the very low penetrance of this phenotype. Therefore, we tested whether double mutants of *pkl* with mutants in close PICKLE homologs *PICKLE RELATED1* (*PKR1*) and *PKR2* [24] had an increased penetrance of this phenotype. We isolated mutant alleles for both genes, located in exon 9 in *pkr1-1* and in exons 9 and 5 in *pkr2-1* and *pkr2-2*, respectively (Figure 1A). Based on the expression of *PKR1* and *PKR2* in isolated homozygous mutant alleles, we concluded that all three alleles are likely to be null alleles (Figure 1B). Neither *pkr1* nor *pkr2* homozygous mutants exhibited significant phenotypic differences to wild-type plants under standard growth conditions (data not shown). However, whereas *pkl pkr2* had a strongly increased penetrance of the *pkl* root phenotype, no increase was observed in the *pkl pkr1* double mutant (Figure 1C). This suggests that PKR2 acts redundantly with PKL in suppressing cell dedifferentiation in the seedling root. This idea is supported by the finding that *PKR2* expression was induced in *pkl* roots (Figure 1D). The *pkr2* mutant did not enhance other aspects of the *pkl* phenotype (data not shown); consistent with lack of *PKR2* expression in other vegetative plant organs (Figure 1D). *PKR2* was strongly expressed in flowers and siliques; however, reproductive development was not disturbed in *pkr2* single and *pkl pkr2* double mutants (data not shown).

### Up- and Down-Regulated Genes in *pkl* Are Enriched for H3K27me3

**Figure 2:**
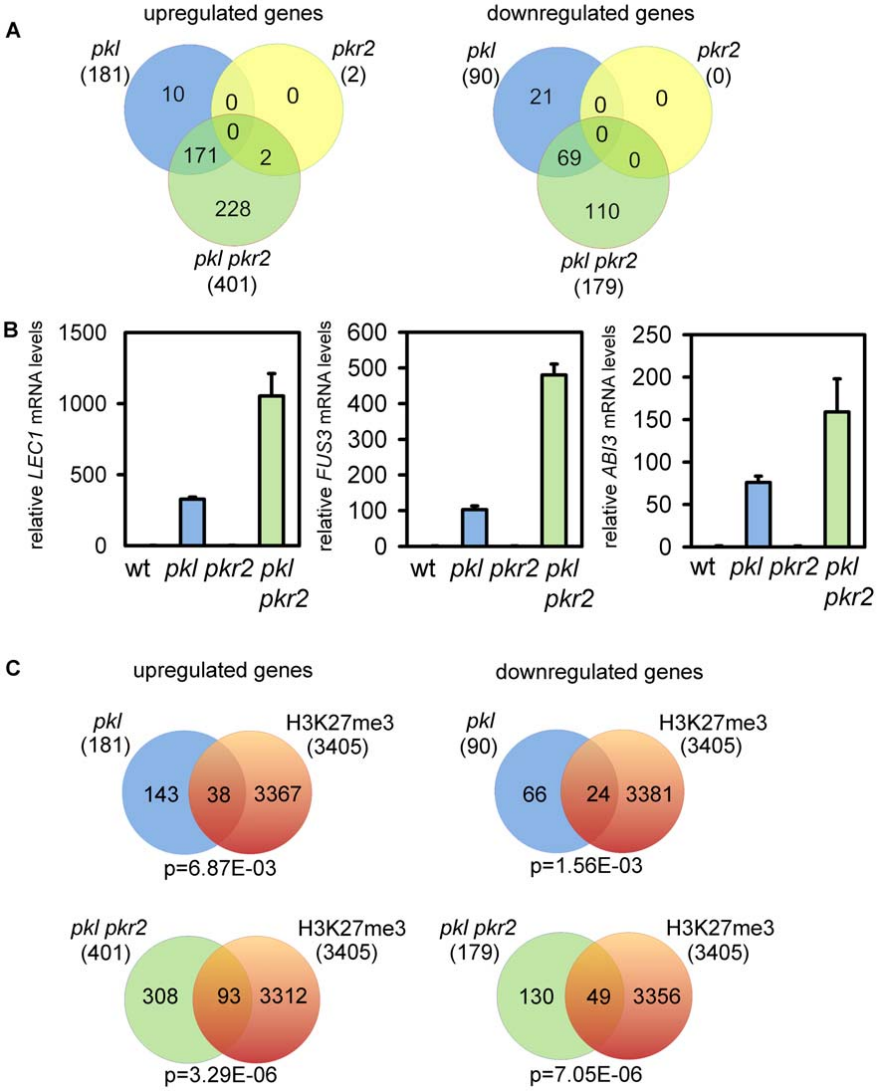
*pkr2* synergistically increases deregulated genes in *pkl* and deregulated genes are marked by H3K27me3. (A)Venn diagrams of up-regulated and down-regulated genes in *pkl*, *pkr2*, and *pkl pkr2* roots. Numbers in parenthesis represent total numbers of up-regulated and down-regulated genes in the respective genotypes. (B) Quantitative RT-PCR analysis of *LEC1, FUS3* and *ABI3* expression in roots of wild-type, *pkl*, *pkr2* and *pkl pkr2* five-day-old seedlings. Error bars, SEM. (C) Venn diagrams of up-regulated and down-regulated genes in *pkl* and *pkl pkr2* and genes marked by H3K27me3 [27]. p-values are based on the hypergeometric test. wt, wild-type.

To investigate the molecular basis of the pickle root phenotype, we profiled transcriptomes of *pkl* and *pkl pkr2* roots at five days after germination. Consistent with the strongly increased penetrance of the pickle root phenotype in the *pkl pkr2* double mutant, we observed a synergistic increase in the number of up- and down-regulated genes in the double mutant (Figure 2A, Table S1). Next we used principal components analysis (PCA) to visualize the relation of *pkl* and *pkl pkr2* mutant roots to wild-type roots, leaves and seeds. PCA was performed on the 4 samples from this study and 11 samples from the AtGenExpress developmental reference data set [25] using expression data of the 611 genes with altered expression in *pkl* or *pkl pkr2* (Figure S1). The primary principle component accounted for 45% of the variation in the data and differentiated between seeds containing embryos and non-embryonic tissue such as roots or leaves. The second principle components accounted for 29% of the variation in the data and differentiated between photosynthetic active (leaves) and inactive (root) samples. Leaf, root and seed samples all clustered tightly in the PCA plot (Figure S1). The *pkl* and *pkl pkr2* samples did not cluster tightly with wild-type or *pkr2* roots but were located between the root and seed clusters indicating a partial change in cell identity from non-embryonic to embryonic fate. The positions of *pkl* and *pkl pkr2* in the PCA plot were consistent with the hypothesis that the *pkr2* mutation enhances the pickle root phenotype. Previous studies revealed that expression of *LEC1* is critically important for cell dedifferentiation and embryonic fate [21],[22],[26]; consistent with this idea we found that *LEC1* as well as embryonic regulators *FUS3* and *ABI3* and other seed-specific genes were synergistically up-regulated in the *pkl pkr2* double mutant (Figure 2B and Figure S2A, S2B).

To explore the connection between PcG proteins and PKL, we tested whether genes that had altered expression in the *pkl* and *pkl pkr2* double mutant were enriched for H3K27me3 [27]. We found a significant overlap with both, up- as well as down-regulated genes in *pkl* and in *pkl pkr2* mutants (Figure 2C, Table S1). It has been reported previously that up-regulated genes in the *pkl* mutant are enriched for H3K27me3 [23], however, the strong enrichment for H3K27me3 among down-regulated genes was unexpected.

### PKL Binds Directly to Genes with Reduced Expression in *pkl* Mutants

**Figure 3:**
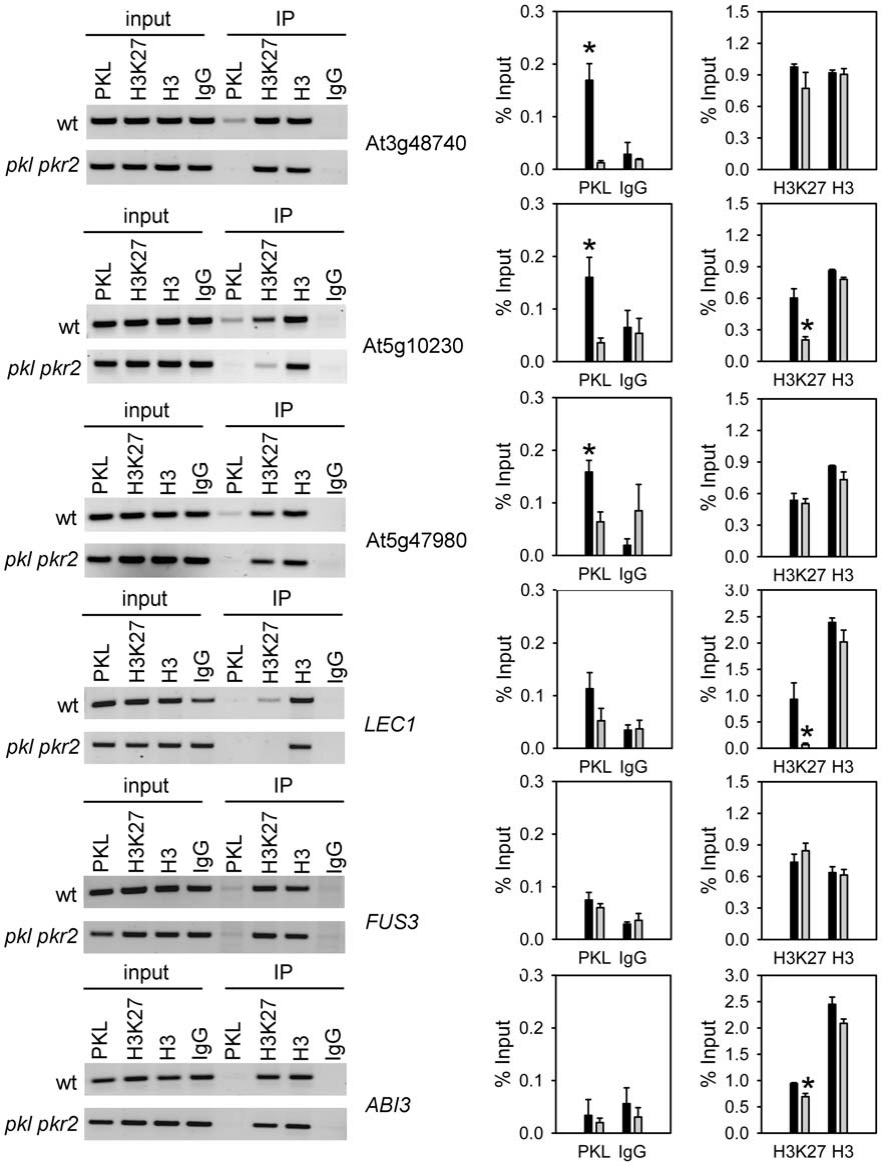
PKL directly binds to genes with reduced expression in *pkl* mutants. ChIP analysis of PKL binding, H3K27me3 and H3 levels at *At3g48740*, *At5g10230*, *At5g47980*, *LEC1*, *FUS3*, and *ABI3* in five-day-old seedling roots. Nonspecific IgG antibodies served as a negative control. ChIP PCR was performed in triplicate, one representative PCR for each locus is shown in the left panels, and quantification of the results show recovery as percent of input in the right panels. Black and gray bars represent wild type and *pkl pkr2*, respectively. Significance of PKL binding (left panels) and reduced H3K27me3 in *pkl pkr2* (right panels) was determined by two-tailed Student's t-test, *P<0.01. Error bars, SEM. IP, immunoprecipitation.

The strong enrichment for H3K27me3 among down-regulated genes prompted us to ask whether PKL was directly required for gene repression, gene activation or whether it had dual function. To address this question we performed chromatin immunoprecipitation (ChIP) using PKL-specific antibodies (Figure S3) and tested binding of PKL to the promoter region of genes with altered expression in *pkl* and *pkl pkr2* mutants. We detected significant PKL binding to three genes that we picked from the top seven down-regulated genes (Figure 3); however, we did not detect significant PKL binding to the up-regulated genes *LEC1*, *FUS3* and *ABI3* (Figure 3), suggesting that PKL is directly required for the activation, but not repression of defined genes.

Consistent with results showing reduced H3K27me3 levels at several up-regulated genes in the *pkl* mutant [23], we detected significantly reduced H3K27me3 amounts at *LEC1* and *ABI3* promoter regions in *pkl pkr2* mutants. No reduction in H3K27me3 levels was observed at the *FUS3* locus (Figure 3), suggesting increased *FUS3* expression is mediated by *LEC1* that was previously shown to activate expression of *FUS3* and *ABI3* [26]. However, we also detected significantly reduced H3K27me3 levels in the promoter region of one of the genes with reduced expression in *pkl* and *pkl pkr2* mutants (Figure 3), suggesting that loss of H3K27me3 is not sufficient for gene activation in *pkl* and *pkl pkr2* mutants.

**Figure 4:**
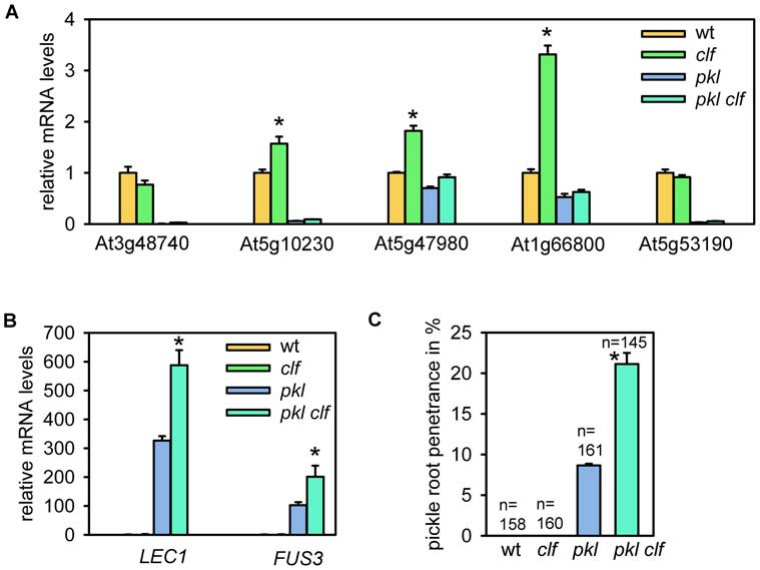
Direct PKL target genes have similar expression levels in *pkl* and *pkl clf* double mutants, while *LEC1* and *FUS3* are synergistically up-regulated in *pkl clf*. Quantitative RT–PCR analysis of (A) *At3g48740*, *At5g10230*, *At5g47980*, *At1g66800*, *At5g53190* and (B) *LEC1* and *FUS3* expression in roots of five-day-old wild-type, *clf*, *pkl*, and *pkl clf* seedlings. Significance of increased mRNA levels compared to wild-type (A) and *pkl* (B) was determined by two-tailed Student's t-test, *P<0.001. Error bars, SEM. (C) pickle root formation was assayed from five-day-old wild-type, *clf*, *pkl*, and *pkl clf* seedling roots. Numbers on top of bars represent total number of scored seedlings. Experiments were performed in triplicates. Significance of increased pickle root penetrance in *pkl clf* compared to *pkl* was determined by two-tailed Student's t-test, *P<0.01. Error bars, SEM. wt, wild-type.

To test this hypothesis, we analyzed expression of confirmed PKL target genes and other genes with reduced expression in the *pkl* mutant in *clf* and *pkl clf* double mutants. It was known that lack of CLF causes strong reductions in H3K27me3 [4]. We found that lack of CLF in a *PKL^+/+^* background led to increased expression for three of five tested genes with decreased expression in *pkl* or *pkl pkr2* (Figure 4A). In contrast, lack of CLF in a *pkl* background did not affect expression of the five tested genes. Thus, increased expression of the test genes upon loss of the repressor CLF requires the presence of PKL. These results support our hypothesis that PKL activity is indeed required for gene activation.

In contrast, *LEC1* and *FUS3* were synergistically up-regulated in *pkl clf* double mutants (Figure 4B), supporting the idea that PKL and CLF are required for repression of both genes. Given that *LEC1* and *FUS3* are not direct target genes of PKL (Figure 3) suggests that PKL indirectly represses target genes by activating a repressor. Consistent with increased expression of *LEC1* and *FUS3* in *pkl clf* mutants, we observed a significantly increased penetrance of the pickle root phenotype in the double mutant (Figure 4C). To summarize, a set of PcG target genes were directly bound by PKL, had *reduced* expression in *pkl* and *pkl pkr2* mutants, and additional loss of CLF did not affect expression. Other PcG target genes were not directly bound by PKL, had *increased* expression in *pkl* and *pkl pkr2* mutants, and additional loss of CLF led to a further increase in expression.

### *pkl pkr2* Mutants Have Reduced Expression of PcG Genes and Reduced H3K27me3 Levels

**Figure 5:**
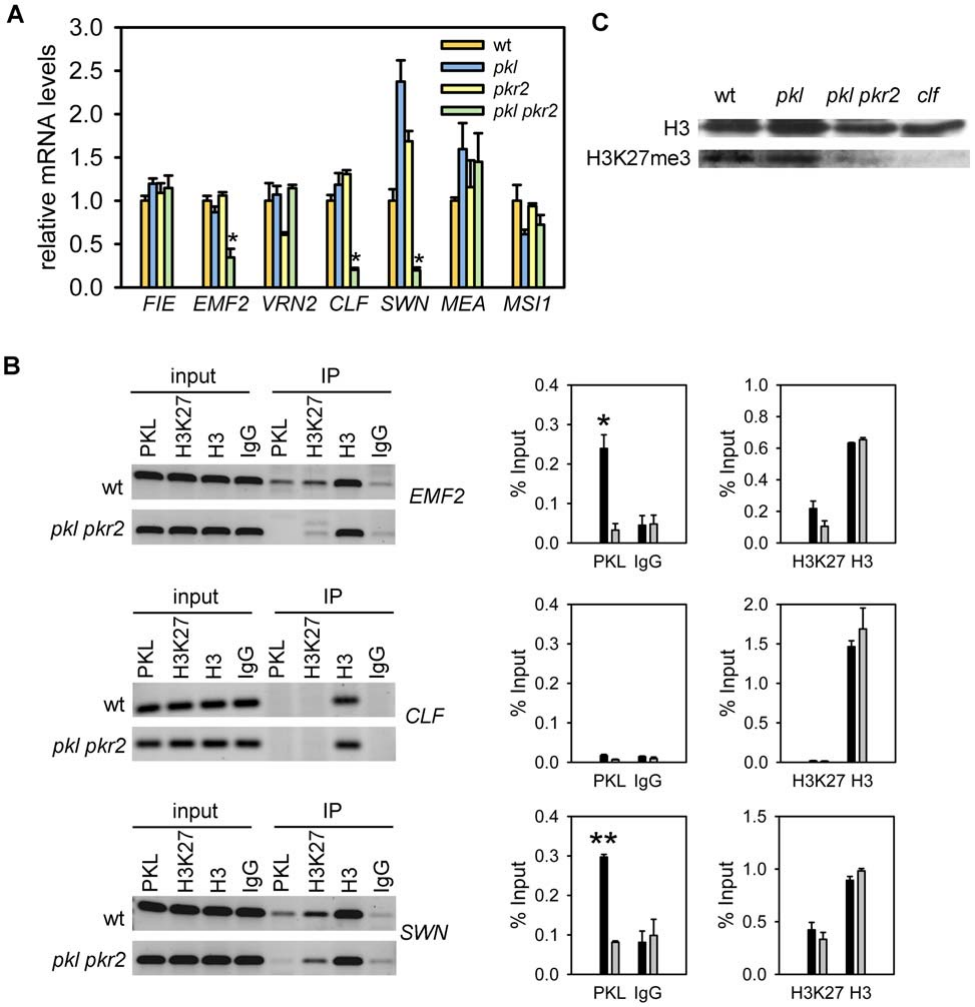
Reduced H3K27me3 levels in *pkl pkr2* are associated with reduced expression levels of direct PKL target genes *EMF2* and *SWN*. (A) Quantitative RT–PCR analysis of *FIE, EMF2, VRN2, CLF, SWN, MEA* and *MSI1* expression in roots of wild-type, *pkl*, *pkr2*, and *pkl pkr2* seedlings. Significance of decreased mRNA levels compared to wild-type was determined by two-tailed Student's t-test, *P<0.001. Error bars, SEM. (B) ChIP analysis of PKL binding, H3K27me3 and H3 levels at *EMF2*, *CLF* and *SWN* in five-day-old seedling roots. Nonspecific IgG antibodies served as a negative control. ChIP PCR was performed in triplicate, one representative PCR for each locus is shown in the left panels, and quantification of the results show recovery as percent of input in the right panels. Black and gray bars represent wild type and *pkl pkr2*, respectively. Significance was determined by two-tailed Student's t-test, **P<0.001, *P<0.01. Error bars, SEM. (C) Western blot anlysis with anti-H3 and anti-H3K27me3 antibodies of wild-type, *pkl*, *pkl pkr2* and *clf* seedling root tissues. wt, wild-type.

A subset of PcG target genes was de-repressed in *pkl* and *pkl pkr2* mutants, and we wondered whether this could be caused by reduced expression of genes for PcG proteins. Therefore, we tested expression of *FIE*, *EMF2*, *VRN2*, *CLF*, *SWN*, *MEA*, and *MSI1* in roots of *pkl* and *pkl pkr2* mutants. Indeed, we detected strongly reduced expression of *EMF2*, *CLF*, and *SWN* (Figure 5A), suggesting that PKL is directly or indirectly required for the activation of PcG protein encoding genes. To distinguish between both possibilities, we performed ChIP analysis and tested binding of PKL to the promoter regions of *EMF2*, *CLF* and *SWN*. We clearly detected binding of PKL to *EMF2* and *SWN* promoter regions, but no binding was detected to the promoter region of *CLF* (Figure 5B) and neither to regions within the gene body (data not shown). Thus, we conclude that PKL is directly required for the activation of *EMF2* and *SWN*, whereas PKL-mediated activation of *CLF* is possibly an indirect effect. We found *SWN* and *EMF2* promoter regions marked by H3K27me3, but no significant enrichment for this mark was detected at the *CLF* promoter, suggesting that PKL is targeted preferentially to PcG target genes. This conclusion was supported by the observation that genes with reduced expression in *pkl* and *pkl pkr2* mutants were also significantly enriched for H3K27me3 (Figure 2C). Previous whole genome analysis of H3K27me3 distribution did not reveal enrichment for H3K27me3 at *EMF2* and *SWN* loci [27], which might be attributed to the use of whole seedlings by Zhang and colleagues (2007) in contrast to the root tissues used here.

**Figure 6:**
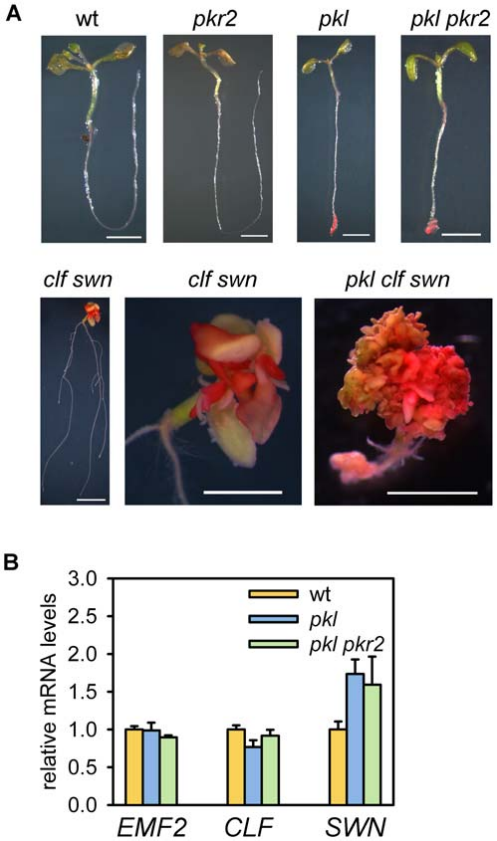
*pkl* enhances the *clf swn* phenotype. (A) Localization of seed storage specific triacylglycerol accumulation in wild-type, *pkl*, *pkr2*, *pkl pkr2*, *clf swn*, and *pkl clf swn* seedlings at 14 days after germination using the lipid staining dye Fat Red. cit_bfScale bars, wt, *pkl*, *pkr2*, *pkl pkr2*, *clf swn*: 0.25 cm; *clf swn* close-up and *pkl clf swn*: 0.1 cm. (B) Quantitative RT–PCR analysis of *EMF2*, *CLF* and *SWN* expression in aerial parts of 14 day old wild-type, *pkl* and *pkl pkr2* seedlings. Error bars, SEM.

Loss of CLF function leads to reduced H3K27me3 levels [4],[28]; therefore, we tested whether reduced expression of genes for PcG proteins EMF2, CLF and SWN in *pkl pkr2* was reflected in reduced H3K27me3 levels. We assayed global H3K27me3 levels and indeed found less H3K27me3 in *pkl pkr2* than in wild-type primary roots (Figure 5C). Previously, we observed induced expression of embryo-specific genes such as *LEC1* and *FUS3* in *clf swn* seedlings [20]. Therefore, we tested whether *clf swn* seedlings developed similar embryonic characteristics like *pkl* [19] and *pkl pkr2* mutants. Indeed, *clf swn* seedlings were clearly stained with the neutral lipid staining dye Fat Red [19], indicating the accumulation of seed storage specific triacylglycerols (Figure 6A). Triacylglycerol accumulation in *clf swn* seedlings was only detected in structures developing from above-ground organs, suggesting that the third E(Z) homolog MEA, which is expressed in wild-type and *clf swn* roots ([29] and data not shown), can compensate the lack of CLF and SWN functions in roots. Triacylglycerols accumulated in *pkl pkr2* seedlings only in primary roots, suggesting that PKL-mediated repression of *EMF2*, *CLF* and *SWN* was restricted to primary root tissues. When testing this hypothesis we found normal expression of *EMF2*, *CLF* and *SWN* in aerial parts of *pkl* and *pkl pkr2* seedlings (Figure 6B). However, lack of PKL function strongly enhanced the *clf swn* phenotype, and *pkl clf swn* triple mutants only formed callus-like tissues that accumulated triacylglycerols (Figure 6A). It is possible that PKL is required for PcG gene activation in primary roots of wild-type plants but also in aerial parts of *clf swn* mutants; reduced expression of other PcG genes would then enhance the *clf swn* phenotype. To summarize, we propose that development of embryonic traits in *pkl pkr2* is a secondary consequence of reduced expression of genes for PcG proteins, resulting in reduced levels of H3K27me3 and faulty expression of embryonic regulators such as *LEC1*, *FUS3* and *ABI3*. This hypothesis predicts a significant overlap of genes up-regulated in *pkl pkr2* and genes up-regulated in *LEC1* overexpressing lines [22]. In agreement with this prediction the overlap of genes up-regulated in *pkl pkr2* and in *LEC1* overexpressing lines was significant (p<1E-15). In contrast, no significant overlap was detected between down-regulated genes of both datasets (Figure S4 and Table S1).

### PKL and PcG Proteins Act Antagonistically on a Similar Set of Target Genes

**Figure 7:**
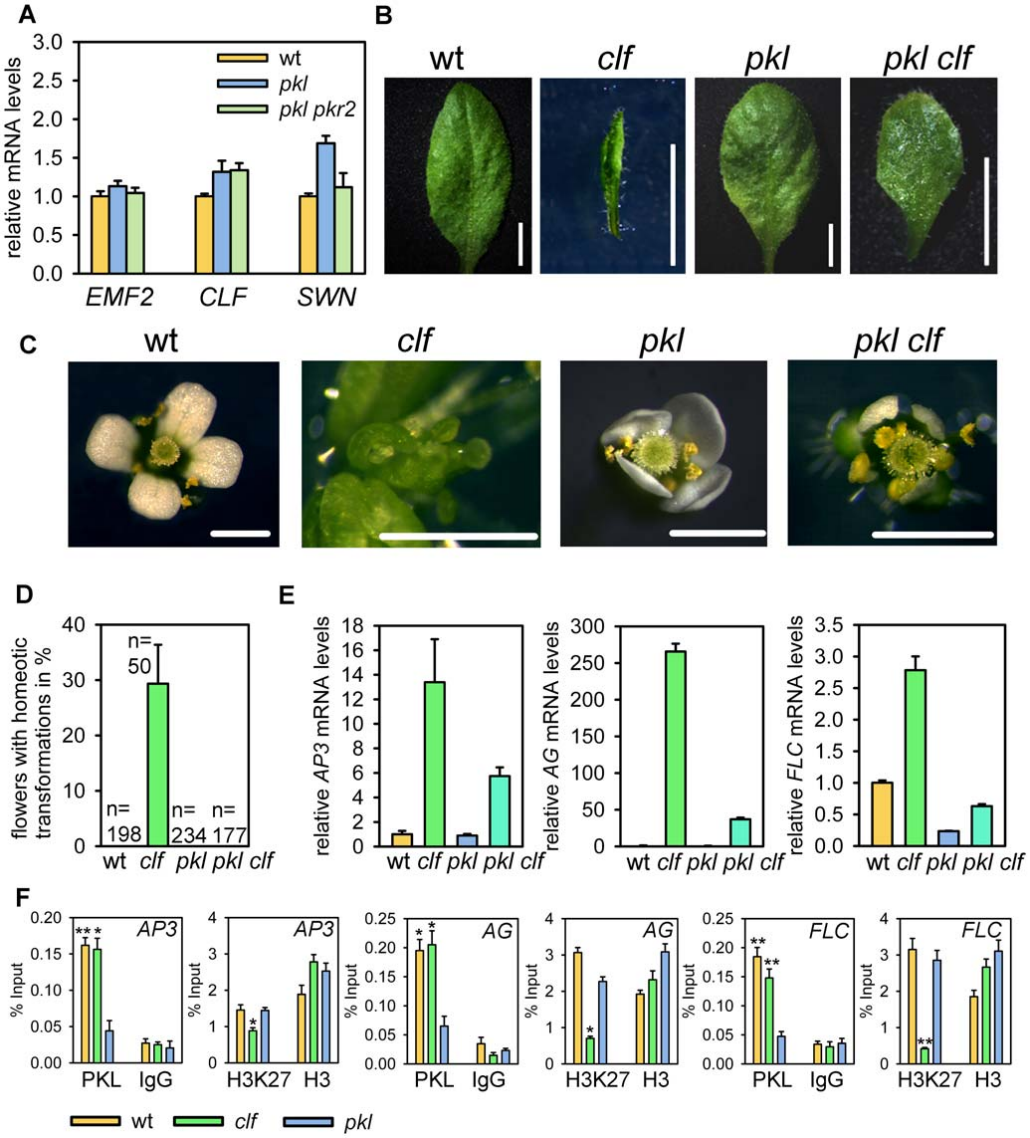
*pkl* suppresses leaf curling and homeotic flower transformations in *clf* mutants. (A) Quantitative RT–PCR analysis of *EMF2*, *CLF* and *SWN* expression in leaves of wild-type, *pkl* and *pkl pkr2*. Error bars, SEM. (B) Leaves of wild-type, *clf*, *pkl*, and *pkl clf*. Scale bars, 5 mm. (C) Flowers of wild-type, *clf*, *pkl*, and *pkl clf*. Scale bars, 1 mm. (D) Quantification of flowers with homeotic transformations in wild-type, *clf*, *pkl*, and *pkl clf*. Numbers on top of bars represent total number of scored flowers. Six individual plants were scored per genotype. Error bars, SEM. (E) Quantitative RT–PCR analysis of *AG*, *AP3*, and *FLC* expression in leaves of wild-type, *clf*, *pkl*, and *pkl clf* seedlings. Error bars, SEM. wt, wild-type. (F) ChIP analysis of PKL binding, H3K27me3 and H3 levels at *AP3*, *AG* and *FLC* in seedlings. Nonspecific IgG antibodies served as a negative control. Quantitative ChIP PCR was performed with four replicates and quantification of the results show recovery as percent of input. Significance was determined by two-tailed Student's t-test, **P<0.001, *P<0.01. Error bars, SEM.

Our transcriptional profiling experiments revealed a significant overlap of genes with reduced expression levels in *pkl* and *pkl pkr2* mutants and genes marked by H3K27me3 (Figure 2C), suggesting that PKL acts as transcriptional activator for PcG protein target genes. To test this hypothesis we analyzed adult phenotypes of *pkl clf* double mutants. Expression of PcG genes *CLF*, *SWN* and *EMF2* was not affected by loss of PKL/PKR2 function in adult leaves (Figure 7A); therefore, lack of PKL function in a *clf* mutant background is expected to suppress at least partially the *clf* mutant phenotype.

There are two prominent phenotypic changes caused by lack of CLF function, (i) *clf* mutants have narrowed and upward curled leaf blades and (ii) *clf* flowers have partial homeotic transformations of sepals and petals towards carpels and stamens, respectively [16]. Both phenotypes were clearly suppressed in the *pkl clf* double mutant. The leaf blade of *pkl clf* plants was flat like the blade of wild-type leaves (Figure 7B) and we did not observe flowers with homeotic transformations in *pkl clf* plants. In contrast, about 30% of *clf* flowers developed homeotic transformations (Figure 7C and 7D). Thus, consistent with our hypothesis that PKL and CLF have antagonistic roles, lack of PKL function largely suppressed the *clf* mutant phenotype. To test whether we could find further molecular support for this hypothesis, we tested expression of the known CLF target genes *AP3*, *AG* and *FLC* [4],[28] in *clf*, *pkl* and *clf pkl* double mutants. All three genes had increased expression levels in *clf* mutants; but expression levels were greatly reduced in the *clf pkl* double mutant (Figure 7E). Finally, we tested whether PKL directly binds to *AP3*, *AG* and *FLC* and performed ChIP analysis of wild-type, *clf* and *pkl* seedlings. We clearly detected binding of PKL to the promoter regions of all three genes in wild-type as well as in *clf* seedlings (Figure 7E). Because PKL binding to *AP3*, *AG* and *FLC* occurred in wild-type as well as in *clf* seedlings while PKL-dependent activation of these genes was only observed in *clf* mutants, it is possible that PKL can activate transcription only in the absence of H3K27me3. In line with this hypothesis we detected significantly reduced levels of H3K27m3 at the three tested loci in *clf* seedlings. Thus, developmental and molecular phenotypes of *clf pkl* double mutants and direct binding of PKL to PcG target genes support the conclusion that PKL is required for the activation of PcG target genes.

## Discussion

### PKL Acts as Transcriptional Activator

The chromatin remodeling factor PKL has been implicated in maintenance of cell identity in plant seedlings by suppressing seed-associated developmental programs [19], [30]–[32], and we found that PKL acts redundantly with PKR2. PKL and PKR2 are homologs of metazoan CHD3/CHD4 proteins [24] that are part of multisubunit complexes with histone deacetylase activity such as the NuRD complex [7],[33]. Therefore, it was suggested that PKL acts as transcriptional repressor and suppresses embryonic regulators like *LEC1* and *FUS3* [6], [23], [30]–[32]. However, previous studies did not detect any effect of PKL activity on acetylation levels, casting doubt on the idea that PKL might be part of a plant NuRD-like complex [23]. Instead, Zhang and colleagues (2008) proposed that PKL is involved in H3K27me3-mediated transcriptional repression, because they found in *pkl* mutants reduced H3K27me3 levels and increased expression for *LEC1*, *FUS3* and several other loci. Nevertheless, the connection between PKL and PcG protein-mediated H3K27me3 remained unclear.

We used ChIP to test binding of PKL to genes that have altered expression in *pkl* mutants and therefore are potential direct PKL target genes. We failed to detect direct binding of PKL to any of the up-regulated genes. In contrast, we detected direct binding of PKL to several of the down-regulated genes. Therefore, we conclude that PKL does not act as transcriptional repressor but as transcriptional activator. Interestingly, Drosophila dCHD3 acts as a monomer and is not part of a NuRD-like complex [10]. Furthermore, Drosophila CHD3/CHD4 proteins dMi-2 and dCHD3 colocalize with active RNA polymerase II on polytene chromosomes [9],[10]. Thus, it is possible that at least some members of the CHD3/CHD4 protein family could have a role in transcriptional activation in animals as well.

### PKL Has a trxG-like Function

PKL can act as transcriptional activator, and genes that are down-regulated in *pkl* mutants are of particular interest. We observed a significant overlap of this set of down-regulated genes with genes reported to carry H3K27me3. All identified direct PKL target genes carry H3K27me3. Thus, one major group of genes activated by PKL consists of PcG protein target genes. Because PKL acts as transcriptional activator of PcG protein target genes, PKL can be considered as a plant trxG protein. A trxG function of a CHD protein is not without precedence, as the Drosophila CHD protein Kismet-L counteracts PcG protein-mediated repression by promoting transcription elongation through recruiting ASH1 and TRX histone methyltransferases [34]. For PKL, this idea is supported by the partial suppression of the *clf* mutant phenotype by *pkl*. In summary, direct activation of PcG genes by PKL explains the down-regulation of many genes with H3K27me3 in *pkl* mutants.

### PKL Is Required for Expression of PcG Proteins that Are Subject of Autoregulation

In addition to down-regulation of H3K27me3-covered genes in *pkl*, we observed up-regulation of many H3K27me3-covered genes as well. This is consistent with earlier observations by others [23]. Because we failed to detect direct binding of PKL to any of the up-regulated genes, we conclude that increased expression of these genes in *pkl* is an indirect effect. We show that this indirect effect is caused by reduced expression of PcG protein encoding genes in *pkl pkr2* roots. We find that in roots *EMF2* and *SWN* loci contain H3K27me3, the hallmark of PcG protein-mediated regulation. Thus, *EMF2* and *SWN*, which code for PcG proteins, are themselves PcG protein targets. Autoregulation of genes for PcG proteins has been observed before in Drosophila [35]. In Arabidopsis, the *MEDEA* (*MEA*) gene, a homolog of E(Z), is repressed by PcG proteins in post-embyronic tissues [36],[37]. Similar to many other PcG protein target genes, *EMF2* and *SWN* require PKL for efficient expression, because in *pkl pkr2* roots expression of both genes is strongly reduced. Expression of *CLF* in *pkl pkr2* roots is reduced as well, but this could be an indirect effect because we detected neither H3K27me3 at the *CLF* locus nor PKL binding to *CLF*. Together, reduced expression of *EMF2*, *SWN* and *CLF* explains the reduced H3K27me3 levels in *pkl pkr2* roots and the de-repression of a number of PcG target genes. As lack of PKL did not prevent increased expression of *LEC1*, *FUS3*, *ABI3* as well as many other PcG target genes, we propose that PKL is required for the activation of only a subset of PcG target genes.

*PKL* and *PKR2* are expressed mostly in the seedling root ([32] and Figure 1C), and loss of cell identity in *pkl pkr2* is restricted to primary root tissues. Thus, PKL and PKR2 function mainly in the seedling root; other proteins might activate PcG protein target genes in aerial organs, possibly other PKL homologs.

### PKL Represses Embryonic Traits Via Maintaining PcG Protein Activity

PcG proteins in plants and animals are master regulators of genomic programs [38]. However, whereas in mammals PcG proteins are required to maintain pluripotency and to prevent cell differentiation, in plants PcG proteins are required to promote cell differentiation by suppressing embryonic development. The PcG proteins CLF and SWN act redundantly and lack of both, CLF and SWN causes cells to de-differentiate and to form callus-like tissues that give rise to somatic embryos [18]. EMF2 is likely part of PRC2-like complexes together with CLF and SWN [18]; EMF2 interacts with both, CLF and SWN in yeast and a weak *emf2* mutant allele resembles *clf* [18]. Mutant studies support the idea that PcG protein function is impaired in *pkl* and *pkl pkr2* roots: First, the *pkl pkr2* and *clf swn* double mutants have similar phenotypes. Both activate the embryonic master regulator *LEC1* (this study and [20]) and both express embryonic traits in seedlings. Second, the *clf swn* mutant phenotype is strongly enhanced in the *pkl clf swn* triple mutant, causing complete transformation of germinating seedlings into callus-like tissues. For several reasons we believe that reduced expression of PcG genes is rather the cause than the consequence of the pkl root phenotype: (i) PcG genes *EMF2* and *SWN* are direct target genes of PKL, (ii) about 35% of *pkl pkr2* mutants undergo transformation to pkl roots, whereas expression levels of PcG genes *CLF* and *SWN* are reduced to 20% of wild-type expression levels, indicating reduced expression levels of PcG genes in roots that do not adopt a pkl phenotype, (iii) in line with the last argument, expression of PcG genes was indeed reduced in *pkl pkr2* roots that did not undergo discernable transformations (data not shown).

*LEC1* is sufficient to induce somatic embryogenesis [21],[22] and development of embryogenic characteristics in *pkl pkr2* roots is likely a consequence of *LEC1* de-repression due to reduced PcG protein activity. LEC1 activates *FUS3* [26],[39], suggesting that increased *FUS3* expression in *pkl pkr2* is a consequence of increased *LEC1* expression. *FUS3* expression increases in *pkl pkr2* despite no detectable decrease in H3K27me3 levels at *FUS3*; this is consistent with previous observations that H3K27me3 is not sufficient for gene silencing [4]. Finally, we conclude that PKL restricts embryogenic potential by regulating expression of genes for PcG proteins that are needed to repress activators of embryonic cell fate such as *LEC1*.

Taken together, our study revealed that the plant CHD3 proteins PKL/PKR2 directly activate PcG protein target genes; thus, PKL/PKR2 have trxG-like functions and counteract PcG protein repressive activities during development. In the future, it will be important to find out how CHD3 proteins and PcG proteins target the same genes and why at certain loci repression dominates and at other loci activation dominates.

## Materials and Methods

### Plant Material and Growth Conditions

All *Arabidopsis thaliana* mutants used in this study are in the Columbia accession. The *pkl* mutant used in this study was the *pkl-1* allele described by Ogas et al. (1997). Mutant alleles *clf-29* and *swn-3* were described previously [17],[18]. *pkr1-1*, *pkr2-1* and *pkr2-2* correspond to WiscDsLox407C12, SALK 109423 and SALK 115303 respectively [40]. Single, double and triple homozygous mutant plants were characterized by PCR (for primers see Table S2). Seeds were surface sterilized (5% sodium hypochlorite, 0.1% Tween-20) and plated on MS medium (MS salts, 1% sucrose, pH 5.6, 0.8% bactoagar). After stratification for one day at 4°C, plants were grown in a growth cabinett under a long day photoperiod (16 h light and 8 h dark) at 23°C. For monitoring pickle root development, plates were incubated in vertical position and the phenotype was scored after 7 days. Experiments were performed in triplicates (three plates per experiment) and each experiment was performed at least three times. 10 day old seedlings were transferred to soil and plants were grown in a growth room at 60% humidity and daily cycles of 16 h light at 23°C and 8 h darkness at 18°C.

### Transcript Level Analysis

Root tips of five-day-old seedlings were harvested and total RNA was extracted using the RNeasy kit (Qiagen). For quantitative RT-PCR, RNA was treated with DNaseI and reverse transcribed using the First strand cDNA synthesis kit (Fermentas). For transcript analysis of aerial tissues, RNA was extracted using Trizol reagent (Invitrogen) and cDNA was synthesized as described above. Gene-specific primers and SYBR green JumpStart TaqReadyMix (Sigma-Aldrich) were used on a 7500 Fast Real-Time PCR system (Applied Biosystems). *PP2A* was used as reference gene. For sequences of primers see Table S2. Quantitative RT-PCR was performed using three replicates and results were analyzed as described [41].

### Anti–PKL Antibodies and Protein Immunoblot Analysis

Anti-PKL antibodies were generated against the C-terminus of the PKL protein (amino acids 1111–1384) by immunizing rabbits with the purified protein. For analysis of PKL protein in wild-type and *pkl-1* mutant plants, rosette leaves were ground in liquid nitrogen and incubated in 2×urea sample buffer (65 mM Tris, pH 6.8, 5% β-mercaptoethanol, 2% SDS, 10% glycerin, 0.25% bromphenol blue, 8 M urea) for 5 min at 70°C. After centrifugation, the protein samples were loaded on a SDS-polyacrylamide gel and analyzed on immunoblots using antibodies against PKL. Equal loading and transfer of proteins was verified by staining the membrane in Ponceau Red solution (0.1% Ponceau S, 5% acetic acid). For analysis of H3 and H3K27me3, nuclear proteins from five-day-old seedling roots were extracted as described previously [42]. Protein blots were first probed with anti-H3K27me3 (Millipore, cat. 07-449) followed by anti-H3 antibodies (Millipore, cat. 07-690).

### Chromatin Immunoprecipitation

Root tips of five-day-old seedlings or aerial parts of ten-day-old seedlings were harvested and proteins were crosslinked in 10 mM dimethyladipimate for 20 min. After washing with distilled water proteins were crosslinked to DNA with 1% formaldehyde for 15 min. ChIP was performed as previously described [20] using antibodies against histone H3 (Millipore, cat. 07-690), H3K27me3 (Millipore, cat. 07-449) and rabbit IgG (Santa Cruz Biotechnology, cat. sc-2027). All tested regions were within 400 bp upstream of the start ATG. For sequences of primers see Table S2. PCR products were analyzed by agarose gel electrophoresis and quantified using ImageJ (http://rsbweb.nih.gov/ij/). Three PCR reactions were used for quantification and results presented as percent of input. Alternatively, gene-specific primers and SYBR green JumpStart TaqReadyMix (Sigma-Aldrich) were used on a 7500 Fast Real-Time PCR system (Applied Biosystems). Quantitative ChIP PCR was performed with four replicates and results were analyzed as described and presented as percent of input [41]. All ChIP experiments were performed at least twice.

### Localization of Triacylglycerols

Whole seedlings were incubated for 1 h in filtered Fat red solution (0.5% Fat Red Bluish in 60% isopropanol), washed three times with water and inspected.

### Microarray Analysis

#### Samples, array design, and hybridizations

Root tips of five-day-old seedlings were harvested, and total RNA was extracted using the RNeasy kit (Qiagen). Three independent biological replicates were analyzed, each replicate containing about 300 seedlings. Labeling and hybridization to the arrays has been described previously [43]. Affymetrix Arabidopsis ATH1 GeneChips® were used throughout the experiment (Affymetrix, Santa Clara, CA). The exact list of probes present on the arrays can be obtained from the manufacturer's website (http://www.affymetrix.com). Analysis was based upon annotations compiled by TAIR (www.arabidopsis.org, version 2007-5-2). Data were deposited into the ArrayExpress database (Accession number E-MEXP-2140).

#### Bioinformatic analysis

Signal values were derived from Affymetrix*.cel files using GCRMA [44].

All data processing was performed using the statistic package R (version 2.6.2) that is freely available at http://www.r-project.org/ [45]. Quality control was done using the affyQCReport package in R. In addition, we calculated coefficients of variation (cv) between replicates as a quantitative measure of data quality and consistency between replicates as described previously [46]. Median cv values for triplicate array sets were between 1.4 and 2.8% demonstrating the high quality of the data. Differentially expressed genes were identified using the limma package in R [47]. Multiple-testing correction was done using the q-value method [48]. Probesets were called significantly differentially expressed when q<0.05. To enrich for biologically relevant changes, only probesets with a minimal fold change of 2 were selected. Data for H3K27me3 target loci were from [27]. The significance of enrichments was estimated based on the hypergeometric test. This test is identical to the one-tailed version of Fisher's exact test, and it is considered to be the most appropriate approach to test overlaps of gene lists [49],[50]. First, the hypergeometric test models a sampling without replacement, where probabilities change during the sampling (in contrast, for instance, to the binomial or chi-square tests). Second, the hypergeometric test is accurate even for small sample size (n<1000). Analysis of tissue-specificity of differentially expressed genes was performed in Genevestigator [51].

## Supporting Information

Figure S1 Principal component analysis. A two-dimensional plot of the first and second principle components of the data showing the relative relationship between the 15 samples based on 611 genes with altered expression in *pkl* or *pkl pkr2*. Expression values were averages of triplicate measurements, and PCA was performed using TMEV (http://www.tm4.org/mev.html).
(2.28 MB TIF)

Figure S2 Seed-specific genes are up-regulated in *pkl pkr2* roots. (A) Microarray analysis of seedling, inflorescence, rosette and root tissues reveals seed-specific expression of genes with increased expression in roots of *pkl pkr2* seedlings. Numbers of microarrays used for this analysis are indicated on right side of panel. (B) Seed-specific genes are synergistically up-regulated in *pkl pkr2* mutants. SLR, signal log ratio. Error bars, SEM.
(4.53 MB TIF)

Figure S3 Anti-PKL antibodies specifically recognize the PKL protein. Western blot analysis with anti-PKL antibodies of wild-type and *pkl* leave tissues. Panel on the left shows Ponceau stained membrane. wt, wild-type.
(1.02 MB TIF)

Figure S4 Venn diagrams of up-regulated and down-regulated genes in *pkl pkr2* seedling roots and seedlings overexpressing *LEC1* (*LEC1 OE* [22]). Numbers in parenthesis represent total numbers of up-regulated and down-regulated genes in the respective genotypes. p-values are based on the hypergeometric test.
(1.07 MB TIF)

Table S1 List of genes deregulated in *pkl* and *pkl pkr2* roots.
(0.09 MB XLS)

Table S2 Primers used in this study.
(0.05 MB DOC)
