# Supplementary figures and images for: CHD3 Proteins and Polycomb Group Proteins Antagonistically Determine Cell Identity in Arabidopsis
Source: PLoS Genet. 2009 Aug 14;5(8):e1000605. doi: 10.1371/journal.pgen.1000605 (PMC2718830; doi:10.1371/journal.pgen.1000605)

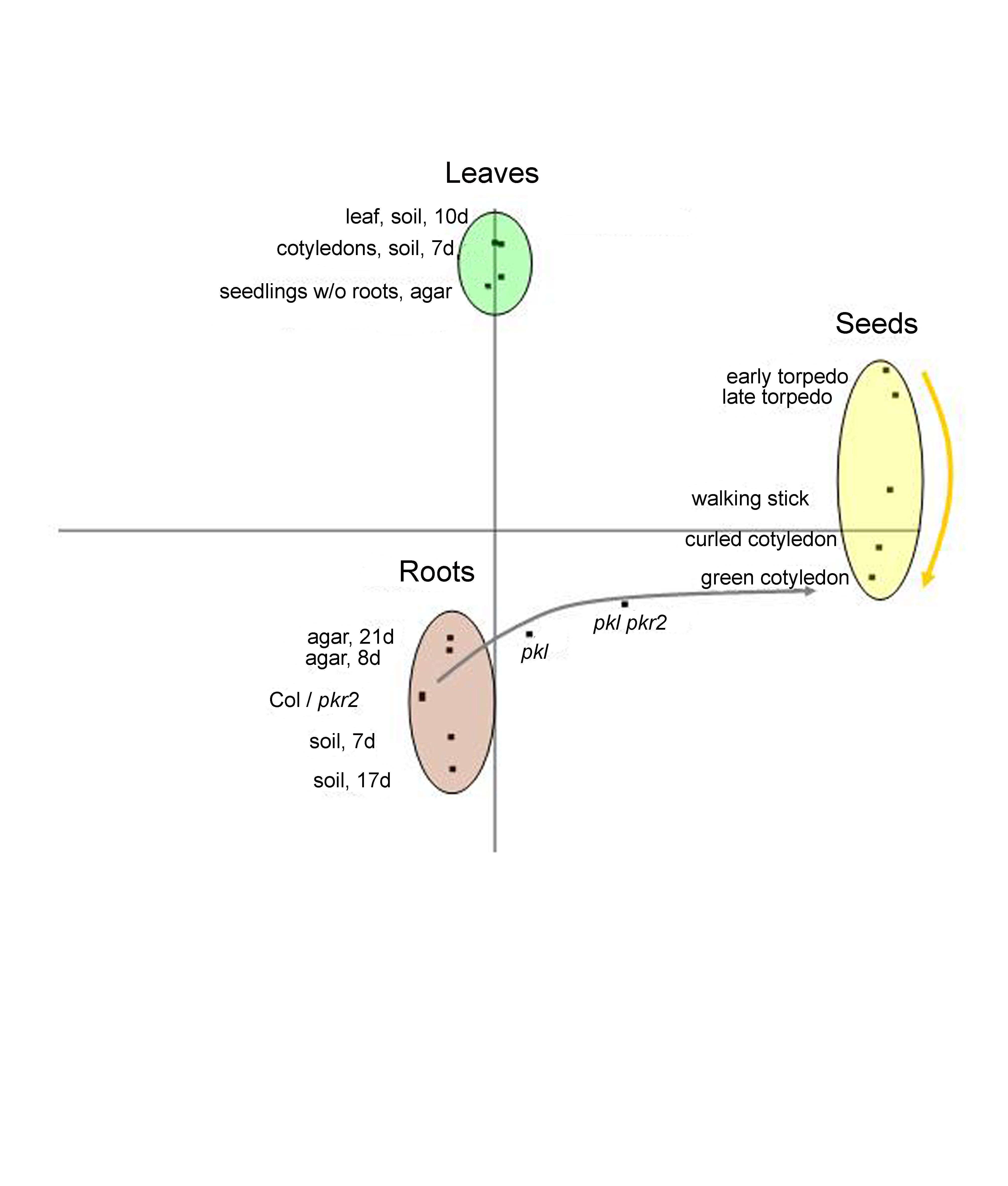

Supplement: Figure S1 — Principal component analysis. A two-dimensional plot of the first and second principle components of the data showing the relative relationship between the 15 samples based on 611 genes with altered expression in pkl or pkl pkr2. Expression values were averages of triplicate measurements, and PCA was performed using TMEV (http://www.tm4.org/mev.html). (2.28 MB TIF) [file pgen.1000605.s001.tif]

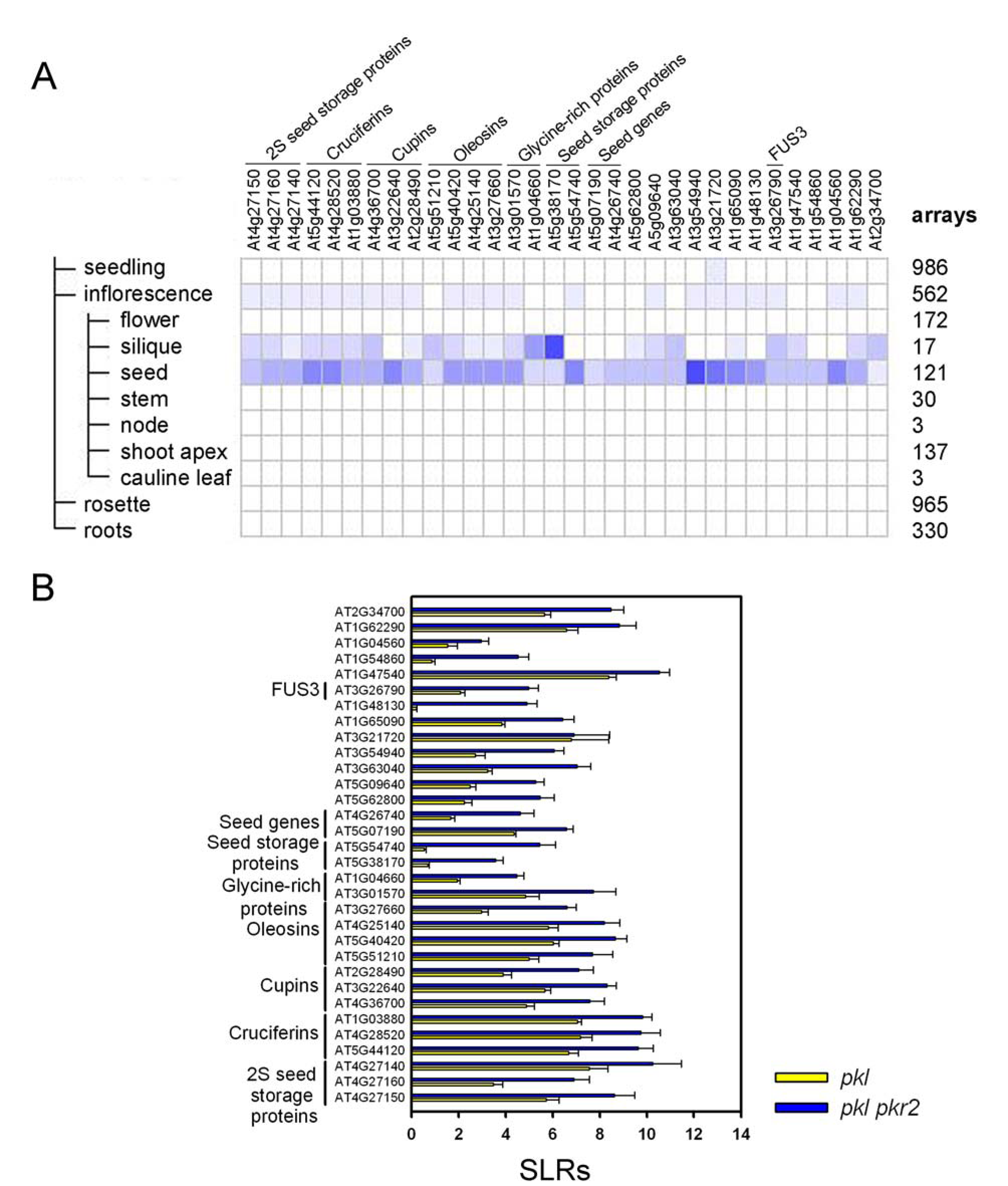

Supplement: Figure S2 — Seed-specific genes are up-regulated in pkl pkr2 roots. (A) Microarray analysis of seedling, inflorescence, rosette and root tissues reveals seed-specific expression of genes with increased expression in roots of pkl pkr2 seedlings. Numbers of microarrays used for this analysis are indicated on right side of panel. (B) Seed-specific genes are synergistically up-regulated in pkl pkr2 mutants. SLR, signal log ratio. Error bars, SEM. (4.53 MB TIF) [file pgen.1000605.s002.tif]

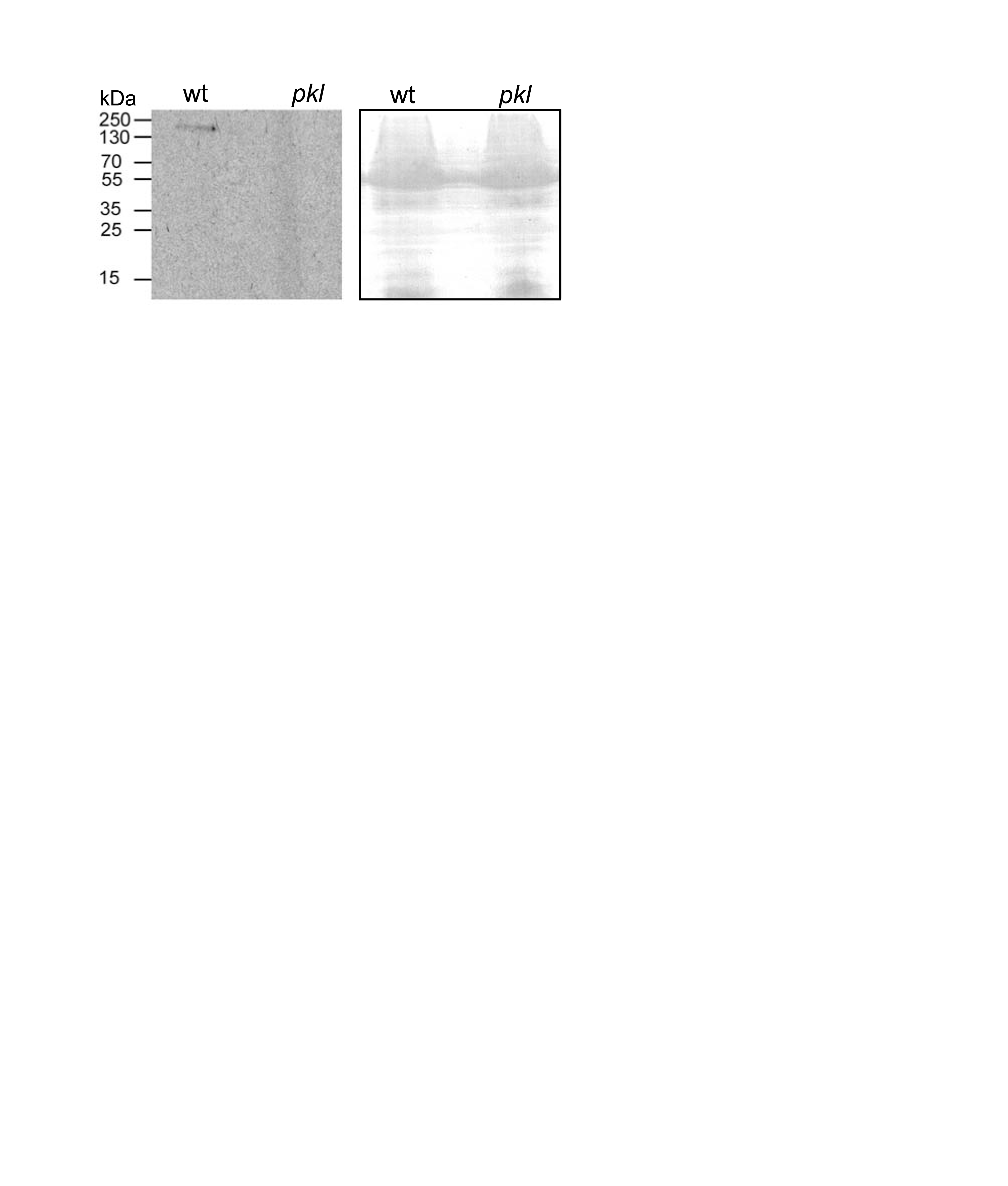

Supplement: Figure S3 — Anti-PKL antibodies specifically recognize the PKL protein. Western blot analysis with anti-PKL antibodies of wild-type and pkl leave tissues. Panel on the left shows Ponceau stained membrane. wt, wild-type. (1.02 MB TIF) [file pgen.1000605.s003.tif]

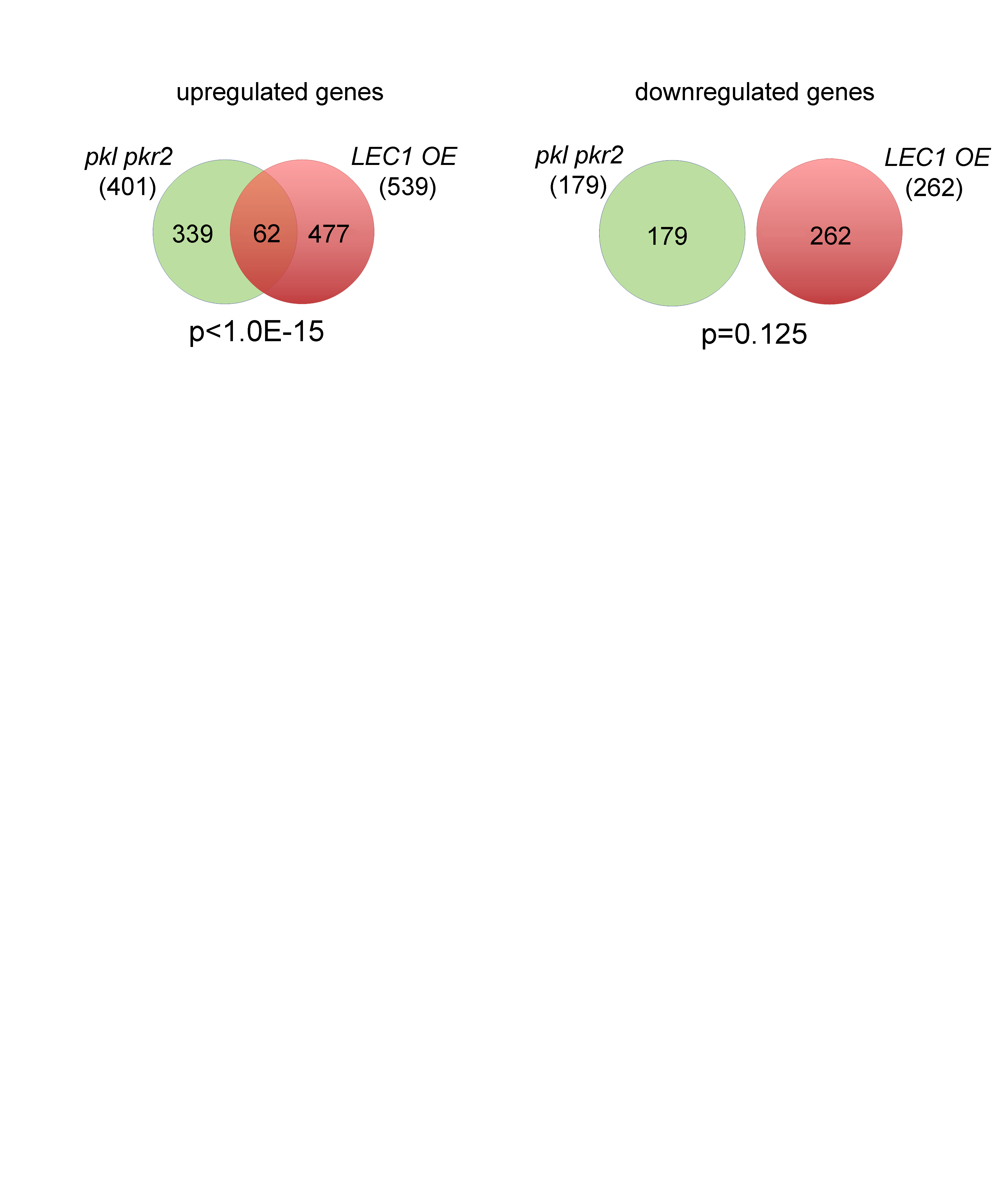

Supplement: Figure S4 — Venn diagrams of up-regulated and down-regulated genes in pkl pkr2 seedling roots and seedlings overexpressing LEC1 (LEC1 OE [22]). Numbers in parenthesis represent total numbers of up-regulated and down-regulated genes in the respective genotypes. p-values are based on the hypergeometric test. (1.07 MB TIF) [file pgen.1000605.s004.tif]
