# Supplementary material for: CHD3 Proteins and Polycomb Group Proteins Antagonistically Determine Cell Identity in Arabidopsis
Source: PLoS Genet. 2009 Aug 14;5(8):e1000605. doi: 10.1371/journal.pgen.1000605 (PMC2718830; doi:10.1371/journal.pgen.1000605)
Supplement: Table S2 — Primers used in this study. (0.05 MB DOC) [file pgen.1000605.s006.doc]

Table S2. Primers used in this study.

| *PKL* qPCR primers | GCTTGTTACATCCATACCAG  TGAATTGTCTTGCCTAGTCC |
| --- | --- |
| *PKR1* qPCR primers | GATAGCAAAGCTAGCCAGCAATG  TTGGTTGAAGACAGGTGGATC |
| *PKR2* qPCR primers | AAGCACGTGATGTTATATGGGAAC  TGCTCATATCAACACGCAAAATG |
| *LEC1* qPCR primers | AAATCCATCTCTGAATTGAACTT  CACGATACCATTGTTCTTGT |
| *FUS3* qPCR primers | GCCAAACAACAATAGCAGAA  TTTCTTGCTTGTATAACGTAATTG |
| *ABI3* qPCR primers | ATGTATCTCCTCGAGAACAC  CCCTCGTATCAAATATTTGCC |
| *At3g48740* qPCR primers | CTTATGAACTTTGGAGGATTCTGTG  TTACCGTCCTGATTATGCTTAGAG |
| *At5g10230* qPCR primers | GAAATCGCTTGTACTAGATCTG  TACCAAGAGCTTTCGAATGTC |
| *At5g47980* qPCR primers | CGATTGGAAACTTACAAGGG  CTCTTCTTTCTCCTTCCTAAACTC |
| *At1g66800* qPCR primers | GCCGTGTTGATAAGGATAATGAG  ATAGGAGTGAACTCAATTCCCA |
| *At5g53190* qPCR primers | TCTCGTCGTTATGAAGAAAGTG  CATATTAGGTGACGCAAGAAAGAG |
| *FIE* qPCR primers | CGTTTCTTCGATGTCTTCGT  ACGACTCTTCCTTATCTTCATCAG |
| *EMF2* qPCR primers | CAGAAGACTGAAGTAACTGAAGAC  AAATTGAGGAGATCGTGGGT |
| *VRN2* qPCR primers | GCAGAAATAACACCAGGAGAC  CCACGGTTTCCATCATTCAG |
| *CLF* qPCR primers | ATTATTCGCATGACCCTTGAG  CATGTCTTGCCTTGATTTCAC |
| *SWN* qPCR primers | CAGGGAATGATAATGATGAGGT  GACCAGCAGACTTTGTAGAG |
| *MEA* qPCR primers | GGTGAGGCACTAGAATTGAGCAGT  CCATAGTCCTGCCCAACCG |
| *MSI1* qPCR primers | CATTTGATAGCCACAAAGAGGAG  TCATCGATCCTGCTAAGGTC |
| *AP3* qPCR primers | CCAGACAAACAGACAAGTGACGTATT  GCTGATATACTCATGAAGCTTGTTGG |
| *AG* qPCR primers | ACGGAATTATTTCCAAGTCGC  GCCTATATTACACTAACTGGAGAG |
| *FLC* qPCR primers | TGTGGATAGCAAGCTTGTGG  TAGTCACGGAGAGGGCAGTC |
| *PP2A* qPCR primers | TAACGTGGCCAAAATGATGC  GTTCTCCACAACCGCTTGGT |
| *CLF* ChIP primers | GTTTCTAATTTACACGCTTCCC  AATCTGAAATGTTGGAGGAG |
| *EMF2* ChIP primers | GGTGGATCTCTAATTTGAACTC  GAAGGTGAAGATTAGGATGG |
| *SWN* ChIP primers | CTCAAACGTAACTGCTATAACC  CATTACATACAAAGAACGCC |
| *LEC1* ChIP primers | TAACTCACTTTCGTAACGCA  GAGGTCTATATCTCTTTCCCA |
| *FUS3* ChIP primers | TGGTTGAGTGTTAGTTTAGTGG  TGGGTTTCAGTGATAGAGATAGAG |
| *ABI3* ChIP primers | GAACAAACTGGAACACATGG  GATCTGAAGTATACGAGATGTG |
| At3g48740 ChIP primers | GATTTGGTAATGGTTTAGCGTG  TCAGTGTTGAAGAGACTCATGG |
| At5g10230 ChIP primers | CCAGCTATCTTGATTTCTTTCC  TGTTGTCTAGATGATGAGGT |
| At5g47980 ChIP primers | CTATCATCCAATTACGTGCT  AAACTCATTGAACGACACCA |
| *AG* ChIP primers | AAGAACTACCCACCAATAACTC  AGAGCAATCTAAAGGTTCAC |
| *AP3* ChIP primers | TTTAGTAACTCAAGTGGACCC  CCCTCTCGCCATATTCTTCTC |
| *FLC* ChIP primers | CTATAGAGTTGCTATGGG  CCAAGTAATAGGTCCACAG |
